# Synthesis of Micro- and Mesoporous Carbon Foams with Nanodispersed Metals for Adsorption and Catalysis Applications

**DOI:** 10.3390/ma16041336

**Published:** 2023-02-04

**Authors:** Roberto García, Elena Rodríguez, María A. Díez, Ana Arenillas, Sara F. Villanueva, Natalia Rey-Raap, Cristóbal Cuesta, María A. López-Antón, M. Rosa Martínez-Tarazona

**Affiliations:** Instituto de Ciencia y Tecnología del Carbono, INCAR-CSIC, C/ Francisco Pintado Fe, 26, 33011 Oviedo, Spain

**Keywords:** carbon foams, sucrose, metal nitrates

## Abstract

This work focuses on carbon foams, whose peculiarity is a predominant open macroporous cellular network that can be provided with tailored texture and morphology by the modification of the preparation process. The goal was to obtain macroporous carbonaceous structures capable of being activated by following a simple thermo-foaming procedure using a few reagents. With this purpose in mind, carbon foams with different textural properties were synthesized from sucrose using two foaming processes: at atmospheric pressure and in a pressurized reactor. Iron and silver nitrates added to sucrose gave rise, after carbonization, to materials with iron oxides and elemental silver particles nano-dispersed in the carbon matrix and promoted microporosity in both cases and mesoporosity in the case of iron nitrate. Iron nitrate also catalyzes the graphitization of the carbon material during carbonization. All these findings show the potential of sucrose thermo-foaming process as a viable and sustainable path to produce versatile carbon materials, capable of being used in various applications.

## 1. Introduction

Developing new functional materials, with tailored structures and properties, is one of the driving forces behind technological progress. In this context, versatile carbon materials, whose characteristics can be modified—by changing the precursor, modifying the preparation method, using additives, etc.—are desirable. Carbon materials are very valuable tools for a wide range of uses. Depending on their characteristics, their applications are as assorted as electronic devices, energy storage, thermal insulation or dissipation, catalysis and water and gas purification, among others [1]. This work focuses on a particular type of carbon material: carbon foams (CFs), whose peculiarity is a dominant open macroporous structure that can be provided with tailored texture and morphology by feedstock selection and modification of the preparation process. The interconnected macroporous substrate ensures an excellent disposition for the recovery and sorption of liquids [2,3], while the incorporation of micropores improves the properties of fixed bed adsorbents [4,5] and catalysts [6].

The synthesis process of CFs usually involves an initial foaming step that gives rise to the macroporous green foam, which is then subsequently carbonized. A good CF precursor must undergo a fluid or pseudo-fluid state during the process of synthesis, which, combined with the production and release of gaseous compounds or the evaporation of an added volatile foaming substance, makes possible the formation of the macroporous structure. Additives, activating agents, heat treatment conditions, etc., provide the final product with different densities, textures, mechanical strength, conductivity, etc., allowing to design the properties of carbon foams for specific applications.

Coal tar or petroleum pitches have been the most widely used precursors for high-performance CFs, in some cases, at a commercial scale [7,8,9]. Moreover, bituminous coals with adequate fluidity have also shown good performance as CF precursors [9,10,11], and synthetic organic polymers, such as phenol-formaldehyde, furfural resin, polyimide and polyarylacetylene, all petroleum-derived chemicals, have been used to produce non-graphitic CFs [2,3,12,13]. However, most of these precursors are not sustainable and generate harmful emissions under heat treatment, requiring strict control. In this scenario, precursors derived from agricultural or residual biomass are proposed as an interesting strategy to synthesize environmentally friendly CF. Tannins, fruit waste or lignocellulosic materials have been tentatively evaluated, but they require complicated synthesis procedures with additional steps, such as liquefaction with alcohols, resinification with formaldehyde or foaming with an appropriate volatile additive [14,15]. Carbohydrates can be also considered suitable precursors for the preparation of CFs. Sucrose is a very abundant disaccharide, containing 42.1% of carbon, that easily undergoes dehydration in acidic conditions, giving rise to a macroporous carbonaceous structure, which can be further carbonized to render a vitreous CF. Carbon foams prepared from sucrose have been gaining interest in recent years, being elaborated and functionalized for very diverse applications such as adsorption and water purification [16,17,18,19,20], catalysis [21], energy storage [22] or electrode manufacturing [23,24].

The most simple procedure for synthesising sucrose-based CFs starts with the concentration of an acidic aqueous solution of sucrose [25,26] to form the viscous caramel through condensation reactions. The caramel is subsequently heated for foaming and setting, with the water vapour generated by condensation reactions acting as foaming agent. The resultant foam is heated in air for annealing and further dehydration and is subsequently carbonized for the release of further volatile compounds and the formation of the carbonaceous matrix, resulting in a high carbon yield and a concomitant enhancement of the mechanical strength [9,27].

The conditions of the manufacturing process can be altered using different types of additives. Metallic nitrates catalyze the condensation reactions while contributing to the foaming by releasing NOx [28,29,30]. Other studies have evaluated the addition of reagents or components to improve specific properties of the final CFs. Boric acid can have a double role in the manufacturing process, acting as a blowing agent and as a source of boron, whose presence in the structure of the foam provides an enhancement of oxidation resistance [31]. Sucrose-derived carbon foams are typically characterized by relatively large structural cells that can confer low mechanical resistance to the material. To address this drawback, different carbonaceous products (active carbon, graphite powder, carbon nanotubes, carbon fibres and others) have been used as reinforcements to improve the strength of the final CFs [32,33,34,35,36,37,38,39], which display increased densities and reduced cell sizes.

This work focuses on preparing CFs from sucrose with the aim of developing a systematic method for obtaining versatile carbon foams that meet specific requirements. The goal is to obtain macroporous carbonaceous structures that can be activated to be used in different applications employing a simple preparation method involving the use of a few reagents that are non-toxic and non-corrosive. The process is intended to be sustainable, cost-effective and in the circular economic context. With these objectives in mind, metallic nitrates have been used as additives to obtain new macroporous materials with different degrees of micro- and mesoporosity and with active metallic species dispersed on their surface. The presence of such metallic species makes these materials potentially suitable for adsorptive and catalysis applications.

## 2. Materials and Methods

Commercial sugar, without any additive, was used as the precursor of the sucrose-based carbon foams SF, rSF1 and rSF2. Citric acid (C) solutions were added into the precursor with concentrations of 0.01 and 0.06 wt.% to catalyze the sucrose hydrolysis. The foams thus prepared were designated as SF(0.01C) and SF(0.06C), respectively. In addition, SF(Fe) and SF(Ag) foams were obtained by adding to sucrose 3 wt.% of Fe(NO_3_)_3_ 9H_2_O and 1.3 wt.% of AgNO_3_, respectively, along with 0.01 wt.% of citric acid. Considering the yields of all the steps, these proportions of nitrates gave rise to concentrations of Fe and Ag in the final foams SF(Fe) and SF(Ag) of the same order, approximately 3%. Metal nitrates have a dual role as foaming and activating agents. To prepare SF(Fe) and SF(Ag), a water solution of each metal nitrate and citric acid was added to sucrose.

The first step in the synthesis of SFs is heating sucrose to be transformed into a plastic state, caramel in the case of sucrose or a dark brown resin in the case of sucrose with nitrates. After cooling, the caramel/resin is heated under controlled conditions to yield the green foam, which will be finally carbonized. The caramel/resin is heated in air with a specific temperature programme and maintained at the selected final temperature. The thermal treatments to obtain the caramel or resin, the green foam and the carbonized foams were evaluated experimentally after being estimated by thermogravimetric analysis.

Thermogravimetric analyses (TGA) were carried out in a Mettler Toledo TGA/DSC1 Star thermoanalyzer. Each sample was evenly distributed in an open alumina crucible and then heated from 30 to 1000 °C at a rate of 5 °C min^−1^ with a holding time of 5 min. Nitrogen was used as an inert gas with a flow rate of 75 cm^3^ min^−1^ to sweep out the volatile products. TGA was also performed in air (75 cm^3^ min^−1^) as an oxidizing gas at a heating rate of 5 °C/min in two ways: from 30 to 600 °C and holding time of 5 min and keeping a low temperature of 250 °C for 60 min. Simultaneously, weight loss and heat flow (in milliwatts) data were registered as a function of temperature, and the first derivative of the weight loss as a function of time (DTG) was calculated.

The temperatures chosen for the preparation of each type of foam are summarized in Table 1. These thermal treatments varied depending on the composition of the precursor, as will be described in the results section, where the conditions to obtain the different carbon foams are discussed. The plastic state (resin) was produced with sucrose and nitrates at temperatures between 120 and 150 °C, while the formation of the caramel with only sucrose and adding citric acid occurred at temperatures of the order of 170–180 °C. In most cases, the heating rate up to the treatment temperature for the green foam was 2 °C min^−1^. Two heating procedures were followed: (i) *free foaming*, performed in an oven with air circulation in an open container, with which the foams SF and SF(C) were obtained, and (ii) *foaming in a pressure reactor*, already described in previous works [10], with which the foams rSF1 and rSF2 were obtained. When Fe(NO_3_)_3_·9H_2_O and AgNO_3_ were used as additives, the free foaming method was employed.

The preparation of the caramel/resin was the key to obtain the foam. This study tested several heating devices (a muffle, an oven with air circulation, a hot plate and a microwave oven) to prepare this plastic state. No differences were observed in the results and the oven with air circulation was chosen. A gentle agitation during the fusion step improved the formation of the foam, and this agitation must not lead to the incorporation of air into the melt. The caramel/resin was prepared in the same container in which the green foam was obtained, a porcelain capsule in the case of free foaming and the reactor itself [10] in the case of pressurized foaming.

In the free foaming procedure, the green foam was prepared from the cold resin in the air circulation oven, heating from room temperature to the selected final temperature for each type of foam (Table 1). Different heating programs were used for the foams with and without nitrates, as the latter favoured the foaming process. After melting, and after the first step of foaming, the product obtained was cooled to room temperature before the subsequent treatment. The caramel/resin and the green foam were prepared in porcelain capsules (100–200 mL) containing amounts of sucrose between 15 and 20 g.

In pressurized foaming, the reactor had a volume of ≈60 mL, and the weight of sucrose used was 10 g. To obtain the foams in the reactor, two procedures were followed. The first one involved melting the sucrose outside the reactor and foaming the caramel/resin following the temperature program shown in Table 1 to obtain foam rSF1. The second involved melting the sucrose in the reactor itself, carrying out a complete temperature program without intermediate cooling, to obtain foam rSF2. The temperature programs were carried out using a fluidized sand bed oven, and the pressure in the reactor reached values of about 40 atm.

In all cases, the green foams were carbonized in a tubular furnace at a programmed temperature (4 °C min^−1^) up to 900 °C, temperature maintained for 2 h under an Ar stream (50 cm^3^ min^−1^).

The porous properties of the samples were determined by nitrogen adsorption isotherms, helium pycnometry and mercury porosimetry. The micro- and mesoporous texture of the foams was analyzed by physical adsorption of N_2_ performed at −196 °C with a Micromeritics ASAP 2420 device. Before measurement, all the samples were outgassed at 180 °C for 12 h under vacuum to eliminate the moisture and condensed volatiles. The specific surface area (S_BET_) was assessed by the Brunauer−Emmett−Teller (BET) equation using the N_2_ adsorption isotherms in a relative pressure range of 0.01 to 0.2. The total micropore volume (V_DR-N2_) was assessed by applying the Dubinin–Radushkevich (DR) equation to the suitable adsorption data in the relative pressure range of 0.005 to 0.17. The mesopore volume (V_Meso_) was determined by the difference between the total pore volume (Vt) and V_DR-N2_. Vt was calculated from the amount of N_2_ adsorbed at a relative pressure of 0.98. The pore size distribution was obtained using the non-local density functional theory (NLDFT) method, with a slit/cylinder model for N_2_ at −196 °C on carbon. However, it should be mentioned that foams are characterized by a large amount of macropores, so this value does not correspond to the real pore volume. Therefore, to accurately evaluate the porous structure of these materials, specifically macropores and mesopores larger than 5.5 nm, both apparent (ρ_Hg_) and true (ρ_He_) densities were determined using a Micromeritics AutoPore IV 9500 device and a Micromeritics Accupyc 1330 device, respectively. The samples were outgassed overnight at 180 °C before the measurements. From these data, the total pore volume (V_Hg_) and the percentage of open porosity (S) corresponding to pore sizes smaller than 12 µm were calculated according to the following equations:

V_Hg_ = (1/ρ_Hg_) − (1/ρ_He_) (1)

S = [1 − (ρ_Hg_/ρ_He_)] 100 (2)

Mercury porosimetry was used as a complementary technique to determine the volume of mesopores and macropores. This analysis was based on Washburn’s intrusion theory. However, it should be considered that only mesopores larger than 5.5 nm can be measured by this technique since this is the lowest limit at which the device can operate [40]. The pore size distribution was obtained from mercury porosimetry by applying increasing pressure to the sample (from 0.005 to 227 MPa).

The powder X-ray diffraction (XRD) patterns of the carbon foams were recorded on a Bruker D8 Advance diffractometer equipped with a Cu-Kα X-ray source (λ = 0.154 nm), operating at 40 kV and 40 mA. Data were collected at 2θ values ranging from 10° to 80°, with a step time of 2 s and a scanning rate of 0.02° s^−1^. The analysis for species identification was carried out with the Diffrac Plus EVA program.

The morphology of the carbon materials was examined by scanning electron microscopy (SEM) using an FE-SEM system Quanta FEG 650 equipped with a S/TEM detector and coupled with an X-ray dispersive energy analyzer Ametek-EDAX with an Apollo X detector.

## 3. Results and Discussion

### 3.1. Foaming

**Figure 1:**
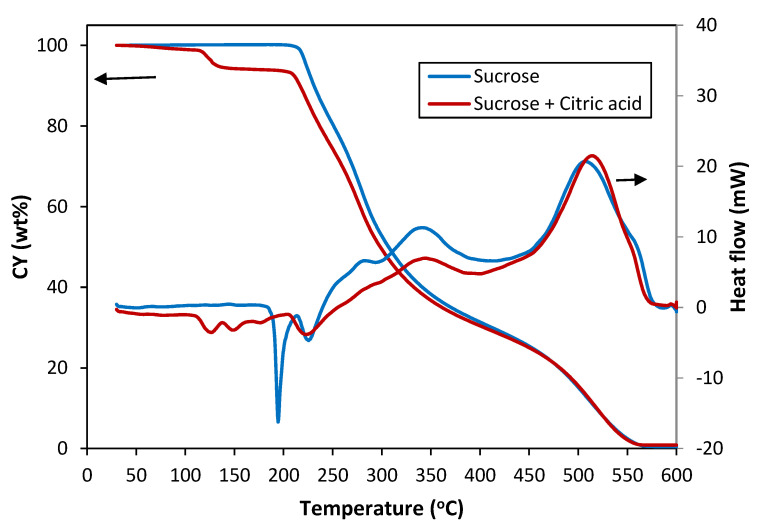
Weight loss (CY) and heat flow curves of sucrose and sucrose with citric acid in air atmosphere.

The application of the heat treatments summarized in Table 1, based on the thermogravimetric analyses, previously published works [25,26] and the experimental observations, gave rise to foams with different characteristics. The objective of the thermogravimetric analyses was to evaluate the behaviour of sucrose with and without different additives in the melting temperature range and the green and carbonized foam formation temperature range without overlooking the experimental conditions for obtaining the foams, which differ from the conditions of the thermal analysis. Figure 1 shows the TG and heat flow curves of solid sucrose without and with citric acid, precursors of SF, rSF1, rSF2 and SF (xC) foams, respectively. From the thermogravimetric analysis under the study conditions, it is observed that sucrose weight remained unchanged up to 210 °C with a sharp endothermic peak at the onset temperature of 185 °C, the temperature at which melting starts. As it is known, after melting, the sucrose molecule partially breaks down to render glucose and fructose (both C_6_H_12_O_6_) [41]. In the range between 185 and 220 °C, complex condensation reactions occurred between these three molecules (the dimer and the two monomers) and their products, giving rise to the formation of the caramel. Some of these reactions seemed to be catalyzed by citric acid, occurring at lower temperatures when the latter was present. For citric acid alone, the maximum decomposition rate occurred at 223 °C, leaving a solid residue of 5.7 wt.% at 250 °C. As the temperature increased well above 250 °C, the combustion of the sample occurred.

**Figure 2:**
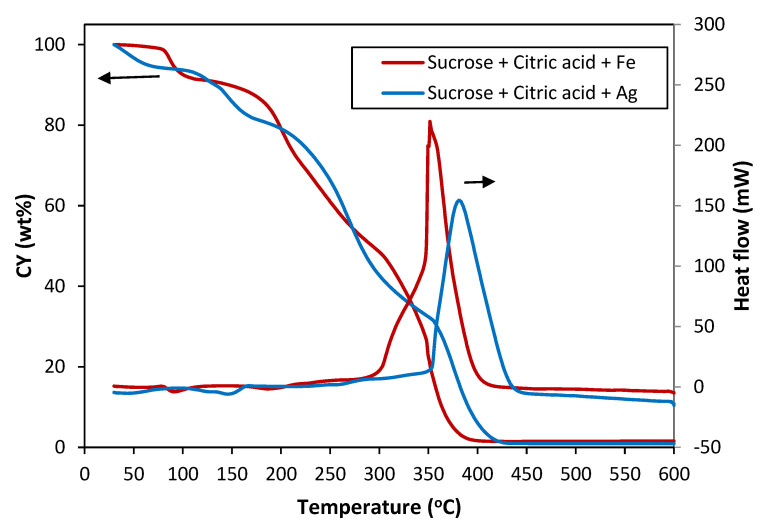
Weight loss (CY) and heat flow curves of sucrose with Fe(NO₃)₃·9 H₂O (Fe)and AgNO_3_(Ag) in air atmosphere.

When Fe(NO₃)₃·9 H₂O or AgNO_3_ were added to sucrose to obtain SF(Fe) and SF(Ag), respectively, the behaviour completely changed (Figure 2). In both cases, complete degradation occurred at temperatures lower than that of sucrose, suggesting some catalytic effect of these metal salts and their degradation products. In the case of Fe(NO₃)₃·9 H₂O, the thermal decomposition was complete at 400 °C, while with AgNO_3_, it occurred at 425–450 °C. Focusing on the temperatures close to the melting point of sucrose and near the formation of the green foam, it is observed that, in both cases, i.e., the addition of Fe(NO₃)₃·9 H₂O and the addition of AgNO_3_, mass losses occurred much earlier than in sucrose. A first transformation entailed a slight loss between 80 and 100 °C and several losses up to 180 °C, the temperature at which sucrose began to melt. Different changes observed between 180 and 250 °C indicate simultaneous reactions to fusion, yielding volatiles as the resin was generated. These results, supported by those observed experimentally, led to lower temperatures or time for the resin formation and modify the green foaming program when the metal nitrates were added to the precursor.

**Figure 3:**
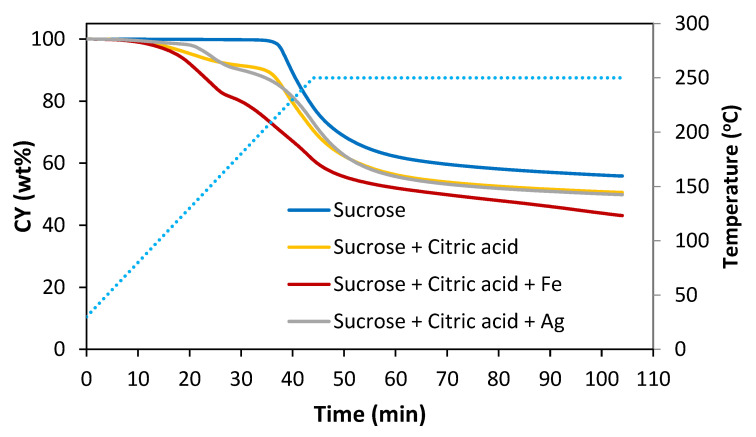
Weight loss curves of sucrose without and with additives in a dynamic step from 30 to 250 °C followed by an isothermal step of 60 min in a controlled air environment.

In an attempt to standardize the procedure for obtaining the foams, 250 °C was chosen as the temperature for obtaining the green foam. At this temperature, in the thermogravimetry tests, the mass loss was complete after 1 h (Figure 3). It was experimentally observed that adding Fe(NO₃)₃·9 H₂O to the precursor resulted in a considerable decrease in the green foam yield (see below, Table 2). For this reason, the heating program was modified for the subsequent experiments conducted with AgNO_3_ to obtain the green SF (Ag).

**Figure 4:**
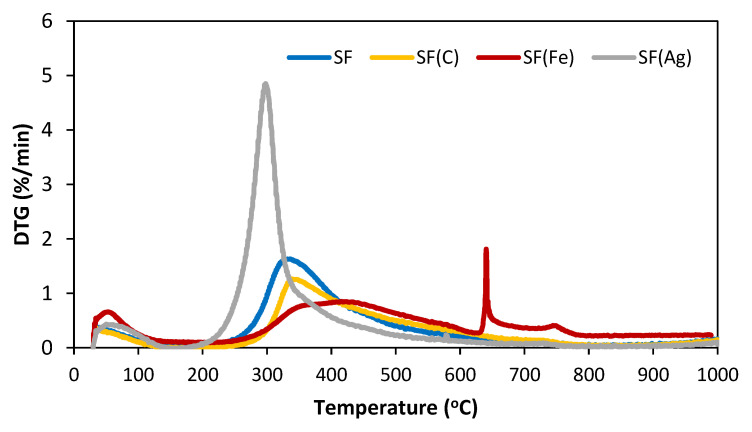
DTG curves of SF green foams in N_2_ atmosphere from ambient up to 1000 °C.

The carbonization temperature of the green foams was chosen from the thermogravimetric analysis carried out up to 1000 °C in a nitrogen atmosphere (Figure 4), looking for the temperature from which the mass remained constant. In all cases, 900 °C was selected since the mass loss that occurred at higher temperatures was lower than 3 wt.%. Figure 4 shows the behaviour of the green foams SF, SF(C), SF(Fe) and SF(Ag) when they were heated in a N_2_ atmosphere at 5 °C min^−1^. Two peculiarities can be observed on the curves of the first derivative of the weight loss as a function of time (DTG): (i) the transformations occurred at lower temperatures in the case of SF(Ag) because this green foam was obtained at a lower temperature (170 °C) than the others (240–250 °C); and (ii) SF(Fe) behaved differently due to the transformations of the iron species at different temperatures higher than 600 °C, which had already been observed in previous works [42]. It should be borne in mind that in the carbonization of the green foam, the iron species were in contact with reducing gases (CO, H_2_) and with carbon acting as a reductant, produced the transformation and reduction in the iron species at 600–700 °C. The reduction reactions can originate elemental Fe when the temperature reaches 900 °C [43], as detailed below.

As already mentioned, the modification of the conditions and the substances added to the precursor lead to materials with different characteristics, which confers high versatility and variability to the products obtained and to those that will be prepared in the future. The drawback, however, is the need to achieve reproducibility in all the described heating processes, mainly in the melting step, for which the conditions must be scrupulously controlled. Bearing this in mind, in this work, the values of the results of characterization of each material, whenever possible, are presented with the uncertainty calculated as the standard deviation of the average value, corresponding to several repetitions obtained under the same conditions.

The yields of the green and carbonized foams are shown in Table 2. The total yield ranged from 23% to 28% in all cases, except for SF(Fe) foam, in which the recovery was lower (16%). The disparity of the results is partially a consequence of the different temperature programs employed for each precursor (Table 1), which, as mentioned above, were selected based on thermogravimetric analysis and preliminary tests. The different behaviour of the precursors, especially the metal nitrates, also had a significant influence.

### 3.2. Morphology of the Foams Identified by SEM

**Figure 5:**
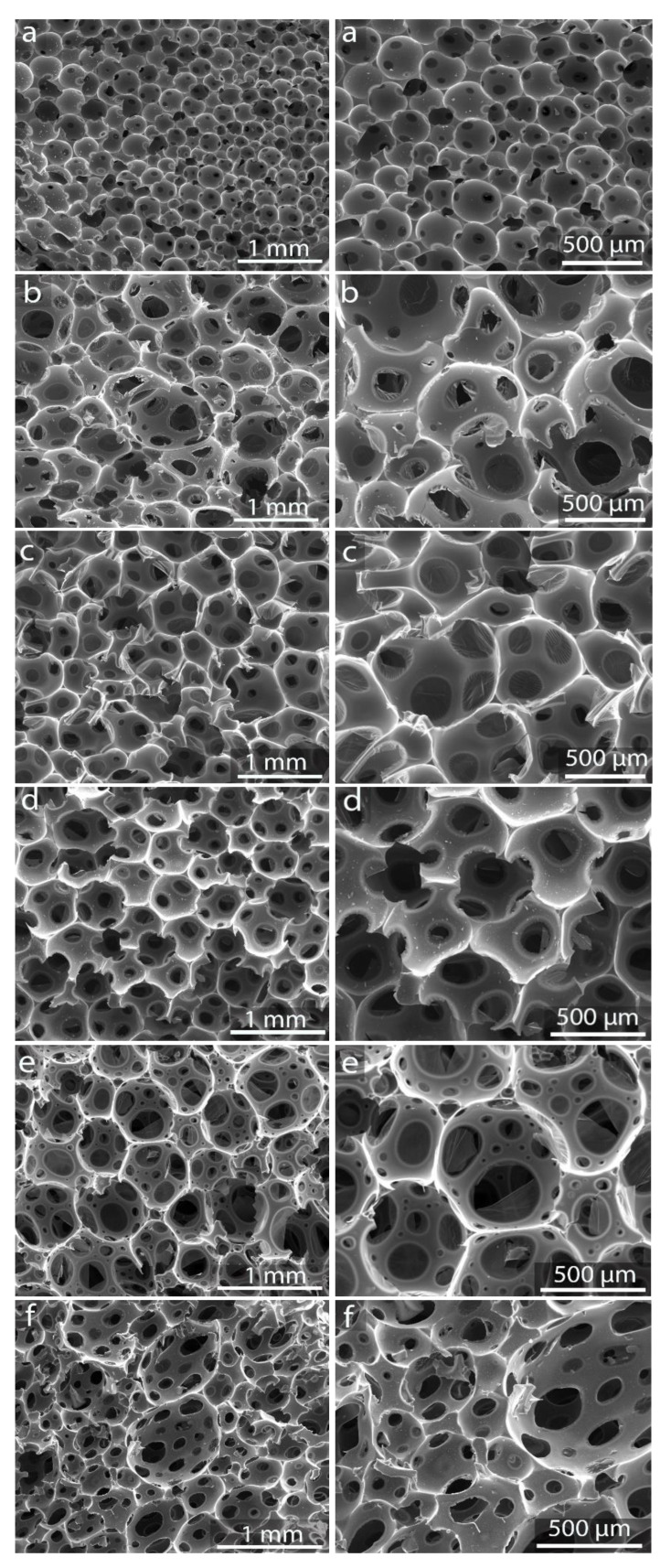
SEM micrographies of rSF1 (**a**), rSF2 (**b**), SF (**c**) SF(C) (**d**), SF(Fe) (**e**), and SF(Ag) (**f**).

SEM analysis illustrates the differences that can be expected in terms of morphology (Figure 5). The SEM micrographs of the sucrose carbon foams showed an open cellular network with cells that display holes or windows in their surrounding solid matter, which is typical of these materials [1,29]. These cells are interconnected through membranes, some of which are broken. This means that the cells are not sealed, eventually allowing the flowing of gases or liquids through the material. A similar morphology has been identified in previous works using different additives [31,35,36,37,44,45]. The boundaries of cells consist of walls that become ligaments when the windows are large enough and struts. In the carbon walls, as will be detailed below, micropores, mesopores, and macropores could appear depending on the precursor, additives and treatment employed. A remarkable feature in all cases is the heterogeneous size of the windows, which also depends on the synthesis conditions. It has been observed that the formation and growth of the bubbles during the foaming process are critical in the final size of the cells and the density of the foam, and the former, in turn, are influenced by the viscosity of the caramel or resin from which the green foam is prepared [28].

Regarding the morphology of the cellular network (Figure 5), differences can be observed in the shape and average diameter of the cells and windows, which is related to the thickness of the walls. The number, size and homogeneity of the cell windows also change. This variability could be exploited by addressing each type of foam to different applications depending on the size and homogeneity of the cells and windows and the density of the material. Comparing the micrographs shown in Figure 5, it can be noted that the greatest heterogeneity in cell and window sizes was observed in the foams prepared with nitrates.

The smallest sizes observed corresponded to one of the foams prepared under pressure in the closed reactor, rSF1, which was significantly different from the other foams. In rSF1, most cells are spherical, with sizes estimated between 100 and 300 µm. The thickness of the walls is overall greater than the size of the windows, which range between 30 and 100 µm, although most are in the lower range. Like most of the obtained foam, this material was prepared from the caramel obtained by melting the sugar in an oven. However, the foam prepared in the pressure reactor starting from sucrose, rSF2, developed a morphology similar to free foaming. rSF2 has similar cell and window sizes to SF. Foams SF and SF(C), obtained by free foaming, have cells of larger sizes, reaching in some cases 1 mm, though most of them range between 500 and 800 μm. The cells were more irregular, showing different degrees of distortion from the spherical shape. The walls looked bigger, with window sizes mostly between 100 and 200 μm, some reaching 500 μm. It can be expected that when foaming is conducted under pressure, swelling is limited, generating foams with smaller cells and windows and higher strength than SF and SF(C) obtained by free foaming, as occurred in the case of rSF1. However, when the caramel is obtained inside the reactor(rSF2), the pressure limitation seems to be compensated by a more significant release of volatiles (those released in both the caramel formation and foaming) and more fluidity in the reaction medium, resulting in a cellular network similar to those of SF and SF(C). These results reinforce the view that the formation of caramel is a primary factor in the development of macroporosity.

The foams containing metals, and essentially those with Fe, show very different morphologies from those described above (Figure 5). They display ligaments rather than walls, and the struts are thinner than the rest of the foams. SEM observations confirm the fragility of the foams obtained with Fe(NO_3_)_3_ 9H_2_O. The cell sizes range from 300 μm to 1 mm, but the particularity that stands out in the morphology of this foam is the enormous heterogeneity of the windows included in each cell, which can range from less than 30 to close to 600 μm. The cells of the foams obtained with silver nitrate have similar sizes to those obtained with iron nitrate, while the window size range is narrower (45–200 μm), with an important proportion of them having sizes smaller than 100 μm.

The bubble generation due to the release of mainly H_2_O vapor, CO_2_, CO and O_2_, derived from the thermal decomposition of sucrose and the carbonization of the green foam, gives rise to the formation of the cells and windows. The presence of nitrates in the sucrose precursor gives rise to the formation of NOx gases which occurs by the decomposition of nitrates according to reactions (R1) and (R2). The increase in pressure inside the bubbles affects the strength of the cell walls. The pores are generated by the rupture of the walls when pressure gradients exceed the resistance of the cell walls [28,29]. The release of NOx contributes to this phenomenon, which could explain the differences between the foams obtained with and without nitrates. In addition, the differences observed in the morphology of the foams obtained with Fe(NO_3_)_3_·9H_2_O and Ag (NO_3_) seem to be due to the different thermal behaviour of the metallic compounds.

4 Fe(NO_3_)_3_ ---> 2 Fe_2_O_3_ + 12 NO_2_ + 3 O_2_ (R1)

2 AgNO_3_ → 2 Ag + 2 NO_2_ + O_2_ (R2)

### 3.3. Metallic Nanoparticles on the Sucrose Carbon Foam Surface

The addition of iron and silver nitrates to the foam precursor was carried out with a double objective. On the one hand, it was intended to improve foaming and promote the activation of foams. It is known that nitrates act as foaming agents and activators, which has been confirmed in this work [28,29]. On the other hand, a second objective was to obtain materials containing active metallic species, which can be addressed to various applications, such as catalysis, adsorption, or the fabrication of electrodes, among others.

**Figure 6:**
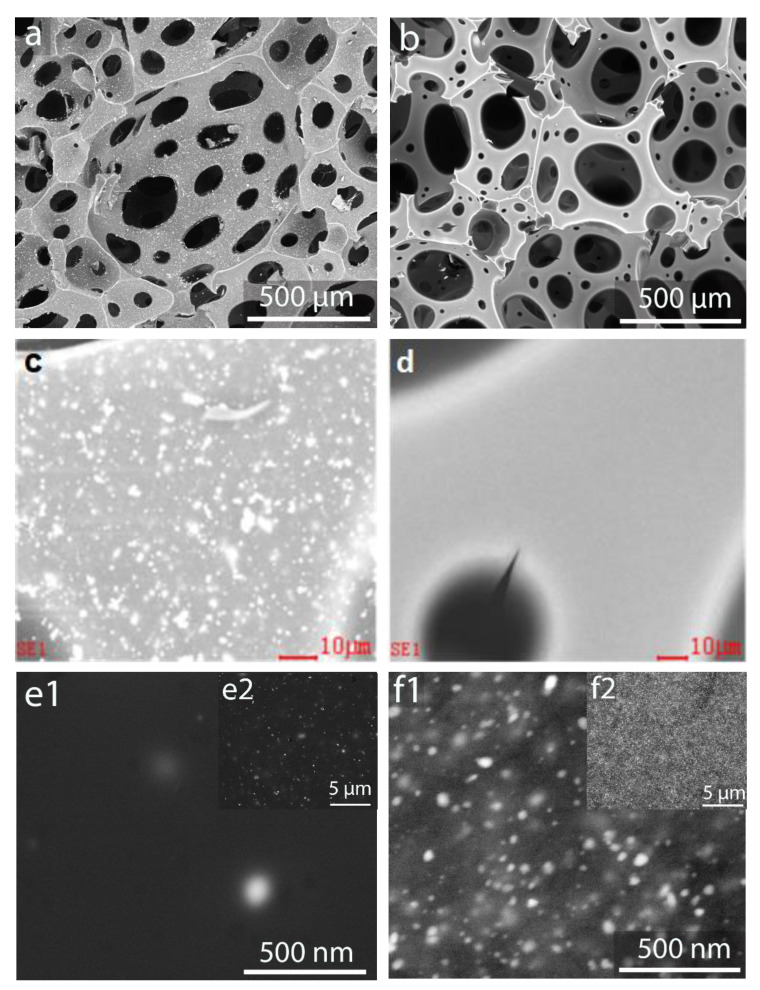
Size and distribution of metal particles in foams SF(Ag) (**a**,**c**,**e1,e2**) and SF(Fe) (**b**,**d**,**f1**,**f2**).

The size and distribution of the metallic species that have been formed on the foam surface were determined by SEM/EDAX analysis. The images obtained by SEM, using a backscattering electron detector (BSED), allowed observing the silver particles deposited on the surface of the foam (Figure 6a), which was more difficult in the case of the deposited Fe species (Figure 6b).

The bright silver species on the foam surface are clearly observed in the SEM micrographs obtained with BSED (Figure 6c). Conversely, the iron species are not visible at the same magnification (Figure 6d), even though they are present. The concentrations of Ag and Fe determined in the cell wall by EDAX analysis reach values of the order of 12% Ag and 10% Fe, indicating that the metals were concentrated on the surface since, in both cases, the proportion of metal added to the whole material was lower (about 3%).

The size and distribution of the metal species deposited on the foam surface can be observed in Figure 6e1,e2,f1,f2, micrographs of silver and iron species, respectively, at different magnification levels. The distribution of metals can be considered homogeneous on the carbonaceous surface (Figure 6e2,f2). It should be noted that the resolution limit of the technique did not allow the clear distinction of the smallest nanoparticles. On the micrographs, the sizes of some of the particles were measured, trying to make the selection representative of the bulk. It can be clearly seen that the Fe-containing particles are smaller than the silver ones. The smallest particles that can be observed were of the order of 10 nm in both cases. However, larger particles or agglomerates were also observed, mainly in the case of Ag species, reaching sizes slightly larger than 200 nm. In the case of Fe, no particles or agglomerates of this size were observed, with the largest size being around 100 nm. It should be noted that most of the nanoparticles of the Ag and Fe species have sizes of smaller than 40 nm. The fact that the metallic species were concentrated on the surface, homogeneously distributed, and with sizes mostly of the order of nanoparticles, makes these materials promising for the abovementioned applications.

### 3.4. XRD of the Sucrose Carbon Foams

**Figure 7:**
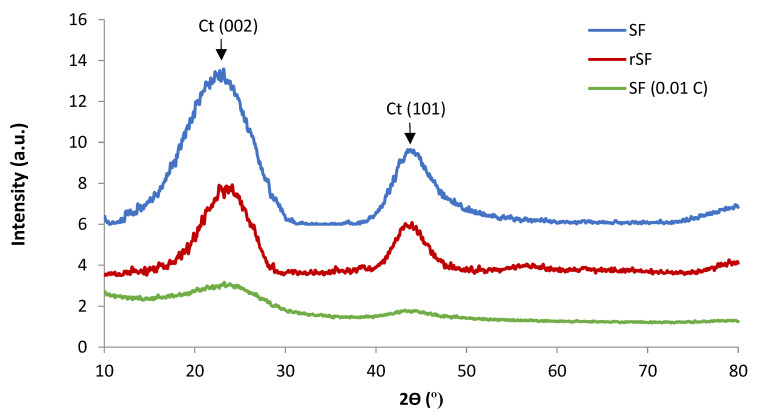
XRD patterns of the carbon foams SF, rSF2 and SF(C). Ct: turbostratic structure.

**Figure 8:**
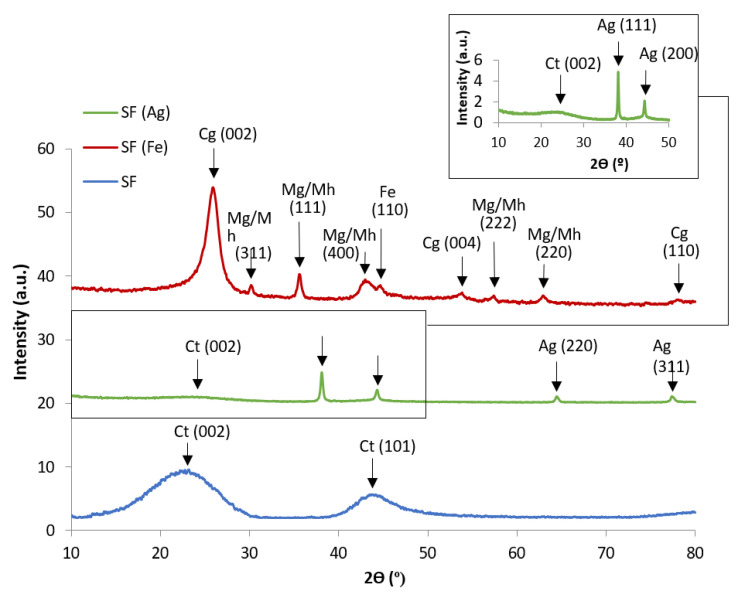
XRD patterns of the carbon foams SF(Fe) and SF(Ag) compared with SF. Cg: graphitic structure; Ct: turbostratic graphite structure; Mg/Mh: magnetite/maghemite; Fe: elemental iron. Inset plot: magnification of the lowest part of SF(Ag) XRD pattern.

The XRD patterns of the carbon foams SF, rSF2 and SF(0.01C) obtained by carbonization at 900 °C (Figure 7) show that these carbon materials have a turbostratic graphite structure (designated as Ct in Figure 7), with broad diffraction peaks at 2θ values of around 24 and 44°, corresponding to the crystalline reflections from (002) and (101) planes. This type of structure is typical of carbon foams obtained with different precursors [25,28,29,38,46,47]. In the case of foams SF(Fe) and SF(Ag), the XRD patterns allow both the identification of the Fe and Ag species originated in the foaming and carbonization processes and the transformations that take place in the carbon material (Figure 8). In the case of SF(Fe), the iron nitrate added to sucrose was chemically transformed during melting, foaming and carbonization at 900 °C, finally giving rise to a magnetic iron oxide, identified as magnetite/maghemite (Mg/Mh) and a small proportion of elemental Fe. In contrast, the silver nitrate added to the sucrose underwent a complete reduction to elemental silver (Ag).

The carbon structure could also be modified in the presence of metal additives. The diffraction peak around 24° was still present in SF(Ag), but significant differences were observed for the carbon foams obtained using iron nitrate (Figure 8). The XRD patterns of SF(Fe) exhibited a well-resolved (002) diffraction peak at 2θ = 26° (designated as Cg in Figure 8). A sharp (002) peak is associated with a high graphitic character compared to the broad diffuse peak observed in amorphous carbon. The porous carbon materials obtained with iron nitrate have an ordered carbon framework due to the graphitization catalyzed by the iron oxide nanoparticles [47].

### 3.5. Macroporosity

Table 3 shows the true density (ρ_He_), determined by helium pycnometry, and the apparent density (ρ_Hg_), the open porosity (Ɛ) and the total pore volume (V_tp_) determined by mercury porosimetry of the sucrose foams. Comparing rSF1, rSF2 and SF, the foams prepared with the same precursor, sucrose alone, the true density of these foams was slightly higher for those samples in which foaming took place in the reactor under pressure. As already mentioned, this can be due to the pressure increase (around 40 bar) derived from the release of volatiles, forced to remain confined inside the reactor and within the reacting material, which means more species are prone to react, giving rise to a more condensed and ordered material. Moreover, the reactor limits the swelling volume of the sucrose, which also contributes to obtain foams with higher true densities. Nevertheless, this is the only similarity between the two foams obtained in the pressurized reactor. In the case of rSF1, obtained from the caramel previously obtained at atmospheric pressure, the non-reactive volatile species generated remain in the reactor and originate pores, giving rise to a material with a pore volume and an open porosity higher than those of the foam prepared at atmospheric pressure. Conversely, in the case of rSF2, the whole manufacturing process (formation of caramel and foaming) was carried out inside the reactor, generating a more significant amount of volatiles that seem to reduce the viscosity of the reacting media [10,11], giving rise to values of apparent density, open porosity and total pore volume more similar to those of SF. A higher homogeneity is also observed in the samples obtained in the pressurized reactor.

More important variations are observed by comparing the foams obtained only with sucrose (rSF1, rSF2 and SF) with those obtained by adding different concentrations of citric acid SF(0.01C) and SF(0.06C) in concentrations as low as 0.01 and 0.06 wt.%. The use of citric acid, which catalyzes the hydrolysis of sucrose producing the inversion to glucose and fructose, modifies the plastic state of the caramel. The presence of citric acid is the only difference in this case; therefore, it is the only variable we can consider. Citric acid catalyzes cross-linking and polymerization reactions of the products of sucrose decomposition and condensation [31], giving rise to foams with higher true density than SF (Table 3). Simultaneously, the enhanced condensation reactions generate more volatiles with a concomitant lower apparent density and higher pore volume and open porosity (Table 3).

As discussed above, the addition of Fe(NO_3_)_3_ 9H_2_O and AgNO_3_ to the sucrose with citric acid significantly modifies the characteristics of the foams SF(Fe) and SF(Ag), giving rise to foams with lower true and apparent densities, and pore volume and open porosity with intermediate values between those of SF and SF(xC). As already mentioned, the creation of the cellular network is a consequence of the release of volatiles that, as seen in thermogravimetric analysis, vary considerably in the presence of nitrates. The metallic nitrates act as catalysts in the condensation reactions of sucrose decomposition products but simultaneously contribute to the release of volatiles with the formation of NOx [29,30]. Moreover, as a consequence of the greater number of volatiles evolved during foaming, the resulting foam showed a higher dilatation. It will also be anticipated that the foams obtained using nitrates display micro- and mesoporosity that account for the total volume of pores, which is not observed in the cases of rSF1, rSF2, SF, SF(0.01C) and SF(0.06C). Comparing the foams prepared in this work with others previously synthesized from coal and coal-sucrose mixtures [11,48], it can be observed that those obtained with sucrose as the primary precursor had, in all cases, lower apparent densities, higher open porosity and larger total pore volume.

**Figure 9:**
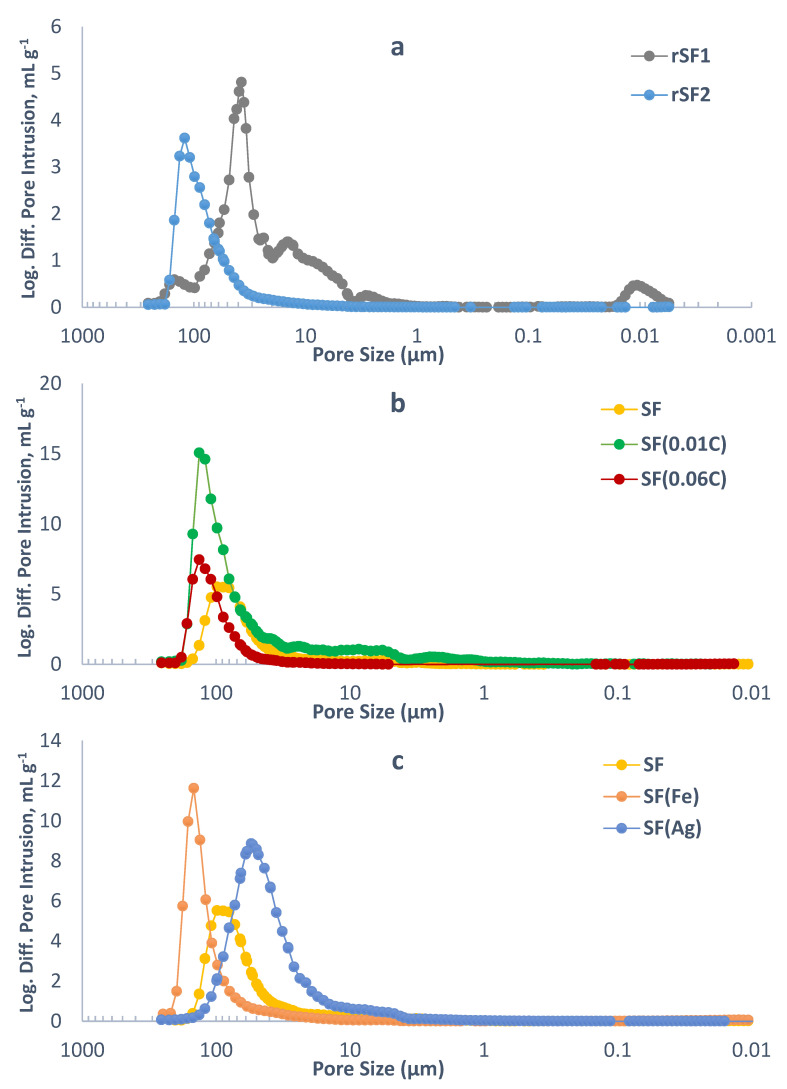
Pore size distribution of: (**a**) carbon foams rSF1 and rSF2 obtained by pressurized foaming; (**b**) carbon foams SF, SF(0.01C) and SF(0.06C) obtained by foaming under atmospheric pressure, and (**c**) carbon foams SF, SF(Fe) and SF(Ag) obtained by foaming under atmospheric pressure.

The pore size distribution estimated by mercury porosimetry of the foams rSF1 and rSF2 is illustrated in Figure 9a, whereas Figure 9b compares the pore sizes of SF, SF(0.01C) and SF(0.06C), and Figure 9c displays those of the foams obtained with the addition of metal nitrates, SF(Fe) and SF(Ag). As already mentioned, several samples were prepared for each type of foam that were independently characterized, having found some deviations in the results. In the case of the maximum pore size, the mean value was 82 ± 16 µm for SF, 82 ± 36 µm for SF(0.01C) and 122 ± 17 µm for SF(0.06C). Figure 9 shows the distributions corresponding to the replicates with the largest sizes, i.e., those included in Table 3. With the limitations marked by the homogeneity of the sample, it can be mentioned that the foams obtained by adding citric acid to the precursor generate larger pores than those that are obtained only with sucrose and that the range of sizes is more extensive, all of which leads to lower apparent densities (Table 3). This finding may be explained by the higher amount of water vapour released due to the catalytic effect of the citric acid on condensation reactions, promoting foam rising. The differences are more significant when metal nitrates are added to the precursor, not only because the maximum pore sizes increase in the case of the foam SF(Fe) and decrease with SF(Ag) compared to SF but also because the size ranges are different.

### 3.6. Surface Area, Micro- and Mesoporosity

**Figure 10:**
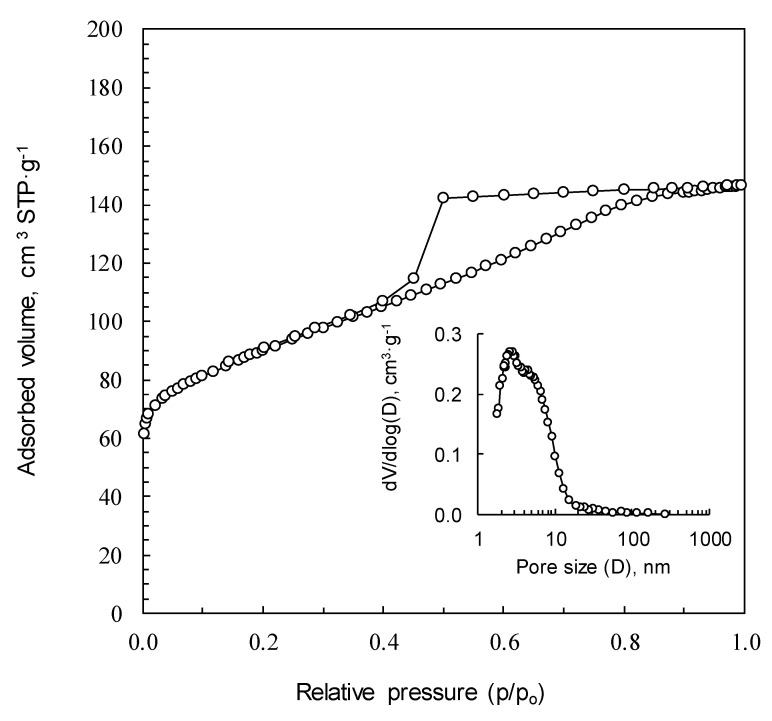
N_2_ adsorption isotherms of SF(Fe). Inset plot: pore size distribution of SF(Fe).

**Figure 11:**
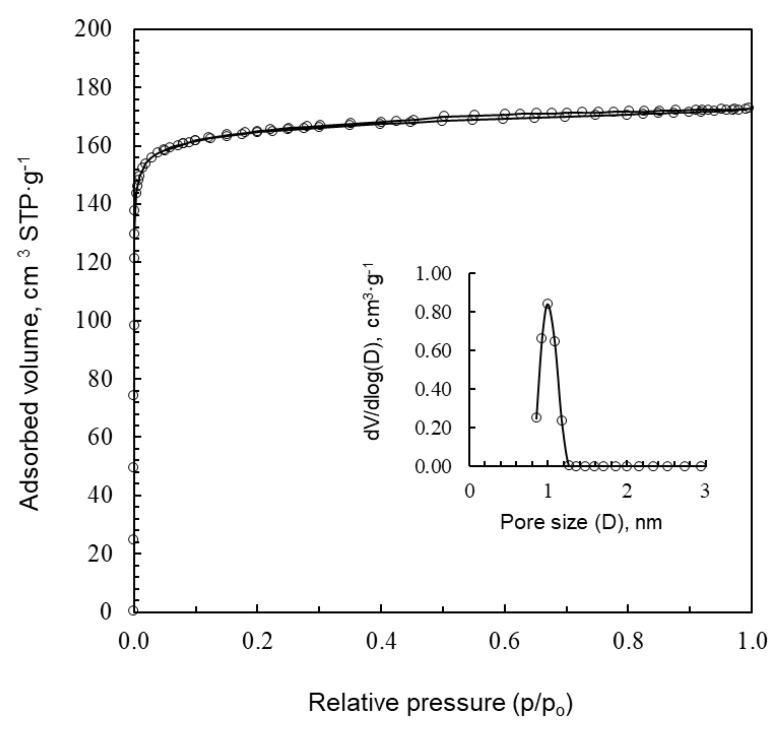
N_2_ adsorption isotherms of SF(Ag). Inset plot: pore size distribution of SF(Ag).

Regardless of the procedure of synthesis, the carbon foams prepared from sucrose without and with citric acid, SF, rSF, SF(0.01C) and SF(0.06C) do not develop micro- and mesoporosity and, consequently, do not display surface area (S_BET_). However, iron and silver nitrates work as activators and the foams obtained with these additives reached S_BET_ areas of 257 and 666 m^2^ g^−1^, respectively. Table 4 shows the calculated BET specific surface areas, the total pore volume (Vt), the micropores volume (V_DRN2_) and the mesopore volume (V_meso_) of all the foams evaluated, and Figure 10 and Figure 11 display the N_2_ adsorption isotherms of SF(Fe) and SF(Ag), respectively. The adsorption isotherm of SF(Fe) is of type IV and exhibits a hysteresis loop associated with capillary condensation, typical of mesoporous materials. The adsorption isotherm of SF(Ag) is entirely different. In this case, it is a type I isotherm, typical of exclusively microporous materials. The total pore volume of both samples is similar, 0.25 and 0.27 cm^3^ g^−1^. However, in the case of the SF(Fe), 56% of the porosity corresponds to mesopores, with sizes between 2 and 20 nm, while SF(Ag) only presents microporosity with sizes between 0.6 and 1.6 nm. The mean pore widths are 5 nm for SF(Fe) and 1 nm for SF(Ag).

These results confirm a different behaviour of iron and silver nitrates when the green foams are carbonized at 900 °C. As already described, the decomposition of silver and iron species during carbonization results in the formation of Ag in its free state and mainly in metal oxides in the case of Fe, generating porosity by activation in the process. In the case of iron nitrate, it has been proposed that not only the carbonization catalyzed by iron oxides generates mesopores, but the particles formed can act as a template to create more mesopores [29,47]. Conversely, the presence of free-state silver in SF(Ag) seems to generate different activation characteristics, giving rise to microporosity [29].

## 4. Conclusions

Carbon foams can be synthesised from sucrose as a precursor by following a simple thermo-foaming procedure. These foams are essentially macroporous cellular materials that can be obtained with different characteristics by changing the manufacturing process: at atmospheric pressure or in a pressurized reactor. The use of citric acid catalyzes the hydrolysis of sucrose and the condensation reactions of the sucrose hydrolysis products, accelerating the caramel formation and modifying the density and the porous structure of the resultant foams.

The addition of iron and silver nitrates to sucrose significantly influences the properties of the final foams. Both compounds catalyzed the synthesis of caramel/resin, reducing the temperature at which it is formed. They also influenced the temperature of foaming, which needs to be significantly lower to prevent the combustion of the carbonaceous network. On the other hand, these additives evolve into iron oxides and elemental silver particles, respectively, nano-dispersed in the carbon matrix. They act as activators and, as a result, the dominant macroporous network of the sucrose foams develops microporosity with both additives and mesoporosity in the case of iron nitrate. Additionally, during the carbonization of the foams, iron nitrate catalyzes an incipient graphitization of the carbonaceous substrate.

From all these observations, sucrose thermo-foaming to obtain carbon materials stands out as a sustainable and highly versatile path leading to carbon materials that can be addressed to very diverse applications. The use of iron and silver nitrates under the described conditions allows the obtaining of new micro- and mesoporous carbon foams, with nanodispersed active metal species on their surface suitable to be employed in adsorptive and catalysis applications.

## Figures and Tables

**Table 1:** Temperature programs used to obtain the sucrose foams (SFs).

	Melting *	Foaming				
	Temp. (°C) (Time at Temp. (h))	Initial Temp. (°C)	Heating Rate 1 (°C min^−1^)	Intermediate Temp. (°C) (Time at Temp. (h))	Heating Rate 2 (°C min^−1^)	Final Temp. (°C) (Time at Temp. (h))
**rSF1**	180 (2)	25	2.0	170 (2)	2.0	250 (2)
rSF2 **	-	25	0.7	150 (0)	0.8	250 (20)
**SF**	180 (2)	25	2.0	170 (2)	2.0	240 (20)
**SF(xC)**	180 (2)	25	2.0	170 (2)	2.0	240 (20)
**SF(Fe)**	150(1)	25	2.0	170 (2)	2.0	240 (20)
**SF(Ag)**	120 (2)	25	2.0	-------	2.0	170 (20)

* Formation of the caramel (rSF1, SF, and SF(C)) or resin (SF(Fe) and SF(Ag)). ** Formation of the caramel in situ, followed by foaming, without intermediate cooling.

**Table 2:** Yields obtained for different carbon foams prepared from sucrose.

	Yield (%)				
	rSF	SF	SF(0.01C)	SF(Fe)	SF(Ag)
Foaming	54 ± 3	48 ± 2	56 ± 3	40 ± 1	86 ± 1
Carbonization	52 ± 1	48 ± 3	45 ± 1	40 ± 3	29 ± 1
Total	28 ± 2	23 ± 2	25 ± 2	16 ± 1	25 ± 1

**Table 3:** True density (ρ_He_), apparent density (ρ_Hg_), open porosity (ε), total pore volume (V_tp_) and maximum pore size of the sucrose foams.

Foam	ρ_He_ (g cm^−3^)	ρ_Hg_ (g cm^−3^)	ε (%)	V_tp_ (cm^3^ g^−1^)	Maximum Pore Size (µm)
rSF1 *	1.96	0.28	85.7	3.1	40
rSF2	2.18 ± 0.08	0.42 ± 0.06	80.7 ± 1.9	1.95 ± 0.31	120
SF	1.71 ± 0.21	0.47 ±0.11	71.1 ± 10.4	1.65 ± 0.64	100
SF(0.01C)	2.14 ± 0.13	0.16 ± 0.02	92.6 ± 0.79	5.94 ± 0.88	135
SF(0.06C)	2.59 ± 0.08	0.16 ± 0.03	93.6 ± 1.4	5.96 ± 1.22	135
SF(Fe) *	1.63	0.25	84.5	3.4	150
SF(Ag) *	1.66	0.19	88.5	4.7	64

* Only one determination.

**Table 4:** BET specific surface areas, total pore volume (Vt), micropores volume (V_DRN2_), and mesopore volume (V_meso_) of the foams.

	S_BET_ (m^2^ g^−1^)	Vt (cm^3^ g^−1^)	V_DR-N2_ (cm^3^ g^−1^)	V_meso_ (cm^3^ g^−1^)
SF	<5	nd	nd	nd
SF(0.010C)	<5	nd	nd	nd
SF(0.06C)	<5	nd	nd	nd
rSF	<5	nd	nd	nd
SF(Fe)	257	0.25	0.11	0.14
SF(Ag)	666	0.27	0.25	0.01

## Data Availability

Data will be made available on request.
